# Insect‒microbe symbiosis-based strategies offer a new avenue for the management of insect pests and their transmitted pathogens

**DOI:** 10.1007/s44297-024-00038-9

**Published:** 2024-12-12

**Authors:** Chao Lv, Yan-Zhen Huang, Jun-Bo Luan

**Affiliations:** Liaoning Key Laboratory of Economic and Applied Entomology, College of Plant Protection, Shenyang Agricultural University, Shenyang, 110866 China

**Keywords:** Insect‒microbe symbioses, Pest control, Sustainable agriculture, Symbionts

## Abstract

With the continuous growth of global agricultural production, pest control has become a critical factor in ensuring crop health and increasing agricultural output. In view of the safety of food and ecology, the development of more environmentally friendly and sustainable approaches for pest management is desirable. All insects are colonized by microorganisms on the insect cuticle or in the body. These resident microorganisms can promote insect fitness, impact the transmission of plant pathogens, or protect insects against natural enemies and adverse environments. Thus, insect‒microbe symbiosis-based strategies provide a new avenue for the management of insect pests and their transmitted pathogens. This review summarizes developments in the field of pest control approaches based on insect‒microbe symbiosis and proposes future directions. First, we introduce insect symbiotic microorganisms and their functions. This review discusses the application of insect-microbe symbiosis-based pest control strategies, including the application of native or engineered symbionts, the utilization of bioactive substances produced by symbiotic microorganisms, and the development of an insect symbiosis disruption strategy. Despite the great potential of this novel pest-control strategy, many challenges remain, such as the stability of symbiotic bacteria, their environmental adaptability, and their impact on non-target organisms. Finally, the review concludes by suggesting future directions, including improving the targeting specificity of symbiotic bacteria, enhancing their environmental adaptability, and developing integrated pest management strategies that combine this means with others to achieve more sustainable and effective pest control.

## Introduction

As the world's population continues to grow, there is mounting pressure to increase crop yields to meet escalating food demands [1]. The burgeoning demands of global agriculture have made pest control a cornerstone of modern farming practices [2]. However, traditional chemical pesticides have adverse effects on the environment and non-target species [3]. This has ignited an urgent quest for eco-friendly and sustainable pest management strategies that can ensure crop health without compromising the integrity of ecosystems [4].

Insect symbiotic microorganisms, which establish lasting interactions with insects, can be found on the insect cuticle, within the gut, in the hemocoel, and even within cells, such as specialized insect cells called bacteriocytes [5–7]. They contribute significantly to the host insect's physiology, growth, reproduction, development and ecological traits, thereby increasing the overall fitness of the insect [8, 9]. A mutualistic symbiosis, in particular, is a type of symbiotic relationship where both the host insect and the microorganism benefit from the association [10, 11]. The application of intricate relationships between insects and their symbiotic microorganisms in the field of pest management has emerged as a promising frontier [12].

This review provides an extensive examination of the symbiotic relationships between insects and bacteria, highlighting their potential use in pest control. This study discusses how such symbiosis can be used to benefit beneficial insects and control pests, thereby reducing the need for chemical pesticides. This review critically assesses challenges such as ensuring the environmental stability of symbiotic microorganisms and preventing unintended effects on non-target species. Finally, future research directions are identified, with the goal of including insect–microbe symbiosis-based pest control strategies in a comprehensive pest management approach. A better understanding of insect‒microbe symbioses could significantly transform pest control strategies in the future.

## Insect symbiotic microorganisms and their functions

Insect symbionts are microorganisms that have established long-lasting and sustained associations with insect hosts [5, 6, 10, 13]. These microorganisms can be located on the insect's body surface, in the gut or hemocoel, or within cells [7]. The functions of symbiotic microorganisms within insects are diverse. These symbiotic relationships can be broadly categorized into commensalism, parasitism, and mutualism [14]. Each type may function depending on certain insect‒microbe symbioses and environmental conditions [15, 16]. Commensalism, observed in bark beetle galleries, involves one species benefiting from the presence of another without causing harm, providing shelter and nutrients to various insects and microbes that coexist with the beetles [17]. Parasitism is a one-sided relationship where one organism, the parasite, gains at the expense of the other, often to the detriment of the host [18]. In contrast, mutualism is a symbiotic relationship in which both organisms derive direct benefits. This type of relationship is particularly noteworthy because it exemplifies mutual benefit and interdependence. In mutualistic symbiosis, the relationships between microorganisms and their host insects are mutually advantageous, with each party providing essential support to the other. For example, symbiotic bacteria can offer nutritional and other benefits to their insect hosts, significantly impacting insect growth, health, and adaptability, which in turn promotes the proliferation and transmission of symbionts [10].

Certain insects, such as those in the order Hemiptera, consume plant phloem but lack the capacity to produce essential amino acids and B vitamins that are required for their growth and development [19–22]. Symbiotic microorganisms aid in the synthesis of these indispensable nutrients, allowing insects to flourish in nutritionally unbalanced environments and thereby broadening their ecological niches [23–25]. For example, tsetse flies *Glossina* and bed bugs *Cimex lectularius*, which consume vertebrate blood deficient in B vitamins, harbor symbiotic bacteria such as *Wigglesworthia morsitans* and *Wolbachia*, which synthesize these vitamins [26, 27]. Similarly, storage pests such as *Lasioderma serricorne* and *Stegobium paniceum*, as well as seed-feeding insects such as the African cotton stainer *Dysdercus fasciatus*, rely on their symbiotic bacteria for B vitamin synthesis [28, 29]. Some insects have multiple symbiotic bacteria that jointly participate in the host's nutritional synthesis. For example, leafhoppers have two symbiotic bacteria that jointly participate in the host's nutritional metabolism; the symbiont *Sulcia* can provide eight essential amino acids, whereas another symbiont, *Baumannia,* can provide two other essential amino acids and a variety of B vitamins [30, 31]. A few facultative symbionts in aphids retain some metabolic pathways similar to those of obligate symbionts, such as the ability to partially compensate for the impact caused by the absence of *Buchnera* in aphids, and may be involved in the synthesis of some amino acids and vitamins [32, 33]. Insect gut microbes, such as microbes in the gut of the African cotton strainer *Dysdercus fasciatus*, which play an important role in maintaining the supply of B vitamins in the body, are also involved in the nutritional metabolism of the host [28].

Symbiotic microorganisms also play a vital role in protecting their host insects from both natural enemies and environmental stress. On the one hand, symbiotic microorganisms protect insects from predators, pathogens, and parasites. The symbiotic bacterium *Pseudomonas* in the rove beetle *Paederus fuscipes* produces polyketide toxins that protect the beetle from predation by natural enemies [34]. Additionally, the symbiotic bacterium *Regiella* in aphids helps defend against the pathogenic fungus *Pandora neoaphidis* [35]. Similarly, *Burkholderia gladioli*, a symbiotic bacterium of the false ground beetle, generates antibiotics and other antimicrobial compounds, thereby safeguarding the host's eggs from various pathogenic microorganisms [36]. In terms of non-biological stress, symbiotic bacteria contribute significantly to the ability of insects to withstand adverse environmental conditions. For example, the symbiotic bacteria *Serratia* and *Rickettsia* found in the pea aphid *Acyrthosiphon pisum* help the insect withstand high-temperature stress [37]. Similarly, aphid-associated bacteria such as *Serratia*, *Hamiltonella*, and *Regiella* improve host resistance to heat [38]. Additionally, mutualistic gut bacteria such as *Burkholderia* strains assist stinkbugs in degrading fenitrothion, thus increasing their resistance to this pesticide [11].

In addition, symbiotic microorganisms can indirectly benefit their hosts by influencing host behavior [39–42] or by modulating plant defensive responses against the host [43]. However, notably, not all symbiotic microorganisms have a positive effect on their hosts. Some symbionts may increase the host's sensitivity to adverse factors, increasing the vulnerability of the host to changes in environmental conditions [44–48]. Thus, the intricate symbioses of the host-microbe highlight the need for a thorough understanding of these interactions to harness them effectively for pest management.

## The application of symbiotic microorganisms in pest control

**Fig. 1:**
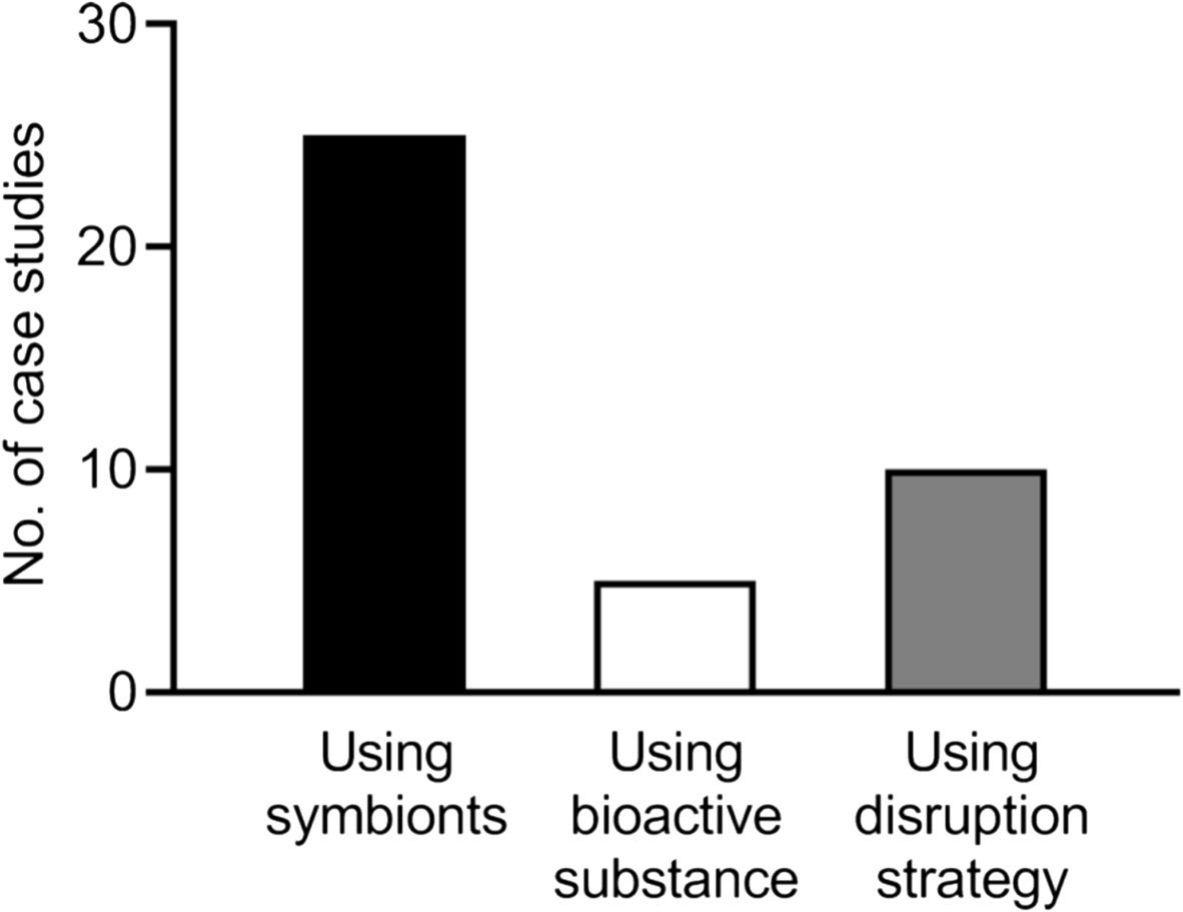
Distribution of pest control strategies based on insect‒microbe symbiosis

**Fig. 2:**
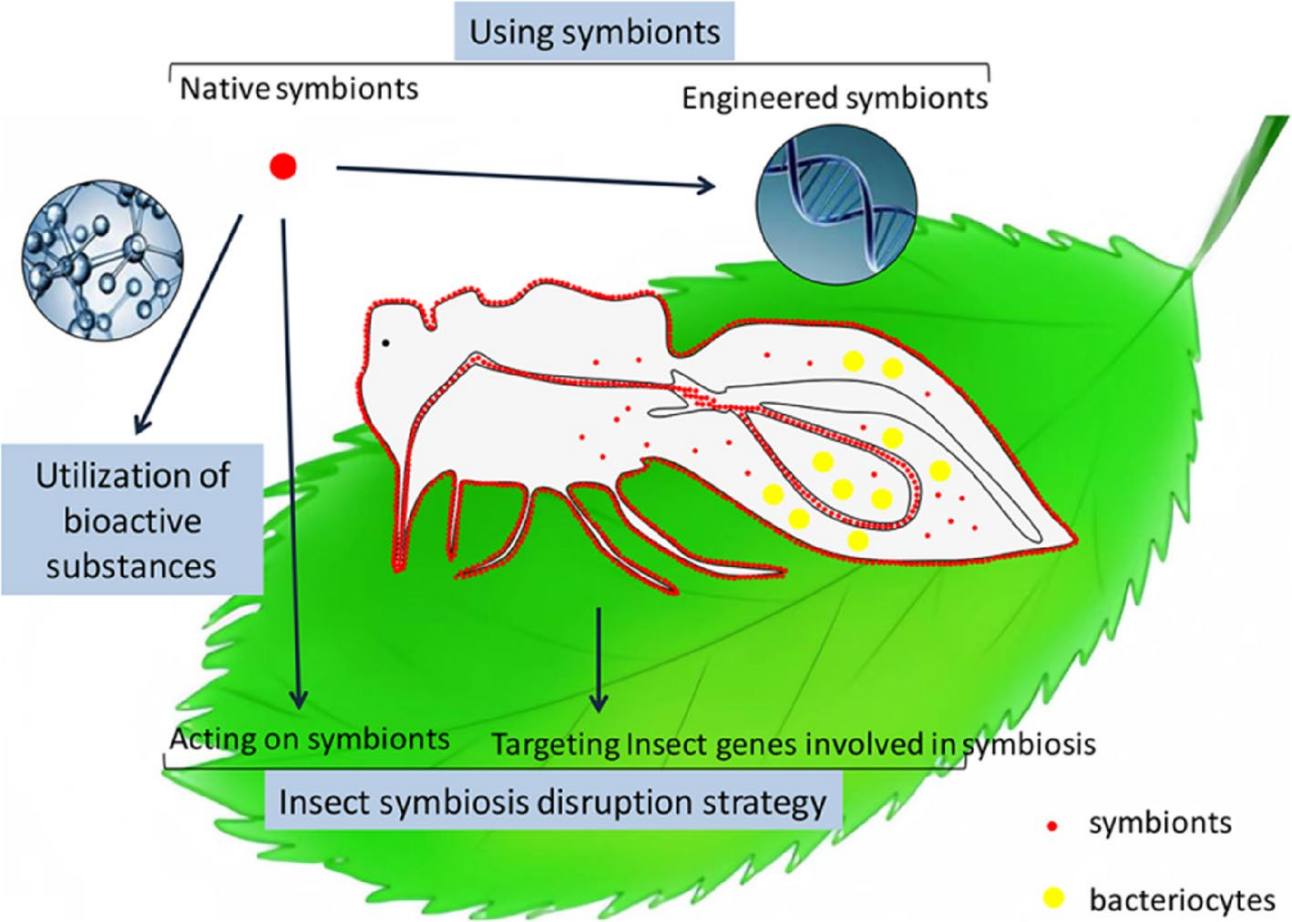
Three key pest control strategies utilizing insect‒microbe symbiosis: the use of native or engineered symbionts to target pest species, the application of bioactive substances produced by symbiotic microorganisms to control pest populations, and the disruption of insect symbiosis to interfere with pest survival or reproduction

In recent years, strategies for controlling pests via symbiotic microorganisms have gained widespread attention (Table 1 and Fig. 2). As of November 7, 2024, a search identified 40 papers on pest control strategies based on insect‒microbe symbiosis, 25 of which focused on the application of native or engineered symbionts in pest control (Fig. 1). For example, *Wolbachia* can rapidly spread into uninfected insect populations by inducing cytoplasmic incompatibility, and it also influences the ability of insects to transmit pathogens [49, 50]. This makes it a promising candidate for pest control, as demonstrated in successful applications for managing mosquitoes and planthoppers. It has been demonstrated that *Wolbachia* can reduce the ability of planthoppers to transmit plant viruses [51] and the capacity of mosquitoes to transmit the human dengue virus [52–54]. Furthermore, the application of *Klebsiella oxytoca*, a gut symbiotic bacterium of fruit flies, as bait in combination with insecticides can significantly reduce fruit fly infestations [55]. In the practice of controlling the brown planthopper *Nilaparvata lugens*, the combined application of antibiotics with insecticides has been shown to significantly reduce the population of Yeast-like symbiotes within the insect, thereby greatly improving the control of the brown planthopper [56].

**Table 1:** Overview of insect–microbe symbiosis-based pest control strategies

Pest control strategies		Hosts insect	Symbionts	Effect on pest control	Reference
The use of native or engineered symbionts	The use of native symbionts	*Mosquito*	*Wolbachia*	Suppress host populations	[51, 60, 61]
		Mosquito	*Wolbachia*	Reduce the risk of dengue	[52–54, 62]
		Fruit fly	*Klebsiella*	Improve sterile male performance	[55, 63–65]
		Brown planthopper	The yeast-like symbiotes	Increase host mortality	[56]
		*Ceratitis capitata*	*Wolbachia*	Suppress host populations	[57, 58]
		Brown planthopper	*w*Stri *Wolbachia*	Block rice virus transmission	[59]
	The use of engineered symbionts	Mosquito	*Serratia*	Inhibit development	[66]
		Mosquito	*Asaia bogorensis*	Inhibit parasite development	[67, 68]
		Mosquito	*Pantoea agglomerans*	Inhibit *P. falciparum* development	[69]
		Honey bee	*Snodgrassella alvi*	Kill parasitic *Varroa* mites	[70]
		*Rhodnius prolixus*	*Rhodococcus rhodnii*	Block pathogens transmission	[71]
		*Homalodisca vitripennis*	*Pantoea agglomerans*	Block *Xylella fastidiosa* transmission	[72]
		Aphid	*Serratia symbiotica*	Affect aphid fitness	[73]
		Tsetse fly	*Sodalis glossinidius*	Reduce *Trypanosoma brucei* transmission	[74, 75]
Utilization of bioactive substances		Mosquito	*Serratia ureilytica* Su_YN1	Kill malaria parasites	[76]
		Locusts	Gut bacteria	Impact host aggregation	[39, 77]
		*Bactrocera dorsalis*	*Klebsiella oxytoca*	Impact host mating behavior	[78]
		Melon fly	Bacterial endosymbionts	Use as attractants	[79]
An insect symbiosis disruption strategy	Acting on the symbiotic bacteria	*Nilaparvata lugens*	Yeast-like symbiotes (YLS)	Increase host mortality	[56]
		*Acyrthosiphon pisum*	*Buchnera aphidicola*	Impact host's arginine synthesis	[80]
		*Oryzaephilus surinamensis*	*O. surinamensis* * Serratia silvanidophilus*	Impact host's cuticle formation	[81]
		*Trialeurodes vaporariorum*	*Arsenophonus and Portiera*	Reduce host survival	[82]
		*Bemisia tabaci*	Gut and bacteriocytes	Impact host's amino acid formation	[83]
	Targeting the host insect's genes	*B. tabaci*	Gut and bacteriocytes	Impact host's amino acid formation	[83]
		*B. tabaci*	*Hamiltonella*	Reduce host survival and fecundity	[84]
		*B. tabaci*	*Portiera*	Reduce host survival and fecundity	[85, 86]
		*B. tabaci*	*Portiera and Rickettsia*	Reduce host fecundity	[87]

The mass rearing and release of sterile male insects is also a technique for pest control via the use of symbiotic microorganisms. For example, adding probiotics from the insect gut, such as *K. pneumoniae*, *K. oxytoca*, *Enterobacter* spp., and *Citrobacter freundii*, to the diet can improve the vitality, development, and mating competitiveness of the Mediterranean fruit fly [63–65].

Genetic engineering of symbiotic microorganisms plays a crucial role in understanding and harnessing insect‒microbe symbiotic relationships, particularly for controlling pests that rely on these symbiotic microorganisms for nutrition, such as cimicid bugs, anopluran lice, tsetse flies, whiteflies, aphids, psyllids, planthoppers, leafhoppers, and various xylophagous beetles and termites [88]. This is achieved by genetically modifying symbiotic bacteria that can colonize and persist within hosts through the conjugative transfer of plasmids from donors to recipients [89]. For example, the bacterium *Serratia*, which naturally resides in the mosquito gut, can be genetically modified to express antimalarial proteins that effectively interrupt the infection and transmission of mosquito-borne pathogens [66]. Similarly, by genetically modifying the bee gut bacterium *Snodgrassella alvi*, it is possible to effectively suppress the parasitism of bee mites [70]. Additionally, in the case of Chagas disease, which is caused by the pathogenic microorganism *Trypanosoma cruzzi* within the cone nose bug, the introduction of antitrypanosomal effector genes into the midgut symbiotic bacterium *Rhodnius prolixus* allows the symbiont to express anti-trypanosomal effector proteins within the midgut of the bug, effectively inhibiting the development of the trypanosome [71]. Although some insect symbiotic microorganisms have been genetically modified under laboratory conditions, it is still necessary to apply these genetically engineered symbiotic microorganisms in field environments to verify their actual effectiveness [85, 90].

## Utilization of bioactive substances produced by symbionts for pest control

As the identification of natural products from traditional sources has declined, scientists have focused their attention on microorganisms from new sources, such as insects [91, 92] (Table 1 and Fig. 2). As of November 7, 2024, a search identified 40 papers on pest control strategies based on insect‒microbe symbiosis, including 5 that explored the utilization of bioactive substances produced by symbiotic microorganisms for pest management (Fig. 1). Insects harbor various types of symbiotic microorganisms that produce a range of bioactive substances. The use of these substances represents a long-term strategy for pest management [7, 93]. For example, a symbiotic bacterium, *Serratia ureilytica* Su_YN1, has natural antimalarial activity in the gut of the major malaria vector *Anopheles sinensis* [76]. This bacterium effectively kills malaria parasites by secreting an antimalarial protein, the lipase antimalarial lipase, revealing the molecular mechanism of its antimalarial activity [76]. Certain symbiotic bacteria produce bioactive compounds that do not directly kill pests but interfere with their behavior and reproduction. For example, the gut symbiont *Pantoea agglomerans* of desert locusts can utilize insects’ digestive byproducts to synthesize precursors of aggregation pheromones, which encourage gregarious behavior in locusts [39]. Conversely, the locust microsporidium parasite can suppress the growth of hindgut bacteria, thereby inhibiting the synthesis of aggregation pheromones and preventing the formation of locust swarms [77]. In the case of the oriental fruit fly *Bactrocera dorsalis* and the melon fly *Bactrocera cucurbitae*, the symbiotic bacteria *Klebsiella oxytoca* and *Citrobacter freundii* can produce significant amounts of compounds such as 3-methyl-1-butanol, 2-phenylethanol, and butyl isobutyrate. These substances act as attractants for female flies, and their proper utilization can be beneficial for pest control [78, 79]. These findings suggest that the use of bioactive substances produced by symbiotic microorganisms is a promising strategy for long-term pest management.

## Development of an insect symbiosis disruption strategy for pest control

Breaking the symbiotic relationship between insects and their symbiotic microorganisms can be achieved not only by acting on symbiotic bacteria but also by targeting host insect genes (Table 1 and Fig. 2). As of November 7, 2024, a search identified 40 papers on pest control strategies based on insect‒microbe symbiosis, with 10 addressing the development of strategies to disrupt insect symbiosis for pest control purposes (Fig. 1). In many insects, the host and symbionts work together to synthesize essential nutrients [80, 81, 83, 94]. This metabolic complementarity is widespread among Hemiptera insects [95–98]. Targeting symbiotic microorganisms of pests, as well as host insect genes to interfere with their symbiotic relationships, is a potential strategy for the control of insect pests and their transmitted pathogens. Agricultural antibiotics, which are secondary metabolites of microorganisms with pesticidal functions, are environmentally friendly and easily degradable and are commonly used for the prevention and control of pests. For example, studies have shown that specific fungicides can significantly reduce the number of yeast-like symbionts (YLS) in the brown planthopper, thereby increasing its mortality rate [56]. Additionally, zhongshengmycin significantly inhibits three symbionts of *N. lugens*—*Pichia guilliermondii*, *Cryptococcus peneaus*, and *P. anomala*, suggesting that this antimicrobial agent may exert its control effects by targeting the symbiotic microorganisms of insects [99]. Furthermore, combining chemical pesticides with antimicrobial agents may represent a promising new strategy for controlling *N. lugens* by inhibiting its symbiotic microorganisms [99]. These findings emphasize the potential of targeting symbiotic microorganisms of pests to control pest populations, providing valuable insights for the development of new pest management strategies. Horizontally transferred genes (HTGs) may complement the missing genes involved in the synthesis of essential metabolites by symbionts [84, 86, 87, 96–98]. The biotin HTGs provide biotin and benefit the fecundity of *B. tabaci* MEAM1 and MED [84, 85]. In addition, whitefly biotin HTGs could complement the missing genes of the symbiont *Arsenophonus* for biotin synthesis in the whitefly *Trialeurodes vaporariorum* [84]. Lysine HTGs (*dapB*, *dapF,* and *lysA*) can cooperate with* Portiera* for lysine synthesis in *B. tabaci* MEAM1 and MED [85, 87, 98]. The vector 2mDNA1, an engineered begomovirus transmitted by the whitefly *B. tabaci*, has been successfully used to silence whitefly biotin and lysine HTGs and thereby repress whitefly performance in both the laboratory and greenhouse [85].

In many insects that act as vectors for viral pathogens, symbiotic bacteria can influence virus transmission [8, 100, 101]. For example, Rice Dwarf Virus can bind to the outer membrane of *Sulcia*, a symbiotic bacterium found in leafhoppers, through a specific interaction between the viral capsid protein and *Sulcia*'s outer membrane protein [102]. Treatment with antibiotics or antibodies targeting this outer membrane protein can disrupt this interaction, effectively preventing the spread of the virus [102].

## Challenges in insect‒microbe symbiosis-based pest control strategies

Insect‒microbe symbioses offer a promising avenue for managing insect pests and their transmitted pathogens, but several challenges must be addressed to maximize the effectiveness of this approach. One major challenge is ensuring the stability of symbiotic bacteria within the insect host. Genetic modifications aimed at enhancing pest control might impact the ability of bacteria to stably colonize the host, potentially leading to a loss of efficacy over time. Research has shown that bacterial persistence and rapid adaptation to changing environmental conditions, such as nutrient fluctuations between the nematode vesicle and insect hemolymph, are crucial for bacterial growth, pathogenicity, and antimicrobial effectiveness [103, 104]. Ensuring the stability of these bacteria across various hosts and environments is therefore a significant hurdle. Additionally, symbiotic bacteria must be able to adapt to a range of environmental conditions [105, 106], including temperature fluctuations, humidity, and exposure to sunlight, to maintain their effectiveness.

Another critical concern is the potential impact on non-target organisms. Ensuring that engineered symbionts do not disrupt the balance of ecosystems by adversely affecting beneficial insects or other environmental factors is essential. For example, specialist algicidal bacteria facilitate the selective control of target algal blooms without impacting non-target species [107]. In contrast, generalist algicidal bacteria might lead to unintended adverse effects on non-target organisms [108, 109].

A further challenge that needs to be considered is the potential for insect pests to develop resistance to symbiosis-based pest control strategies, particularly those involving genetically modified symbionts. Like with conventional chemical pesticides, the repeated use of genetically engineered symbionts can create selective pressure on pest populations, driving the evolution of resistance. One of the most likely mechanisms of resistance is a mutation in the genetic sequence recognized by the nuclease or other molecular tools used to engineer the symbionts. Such mutations could prevent the target site from being cleaved while still allowing the gene to function normally in the insect. These changes may pre-exist in the population, arise through spontaneous mutations, or be induced by the nuclease through end-joining repair mechanisms, and once resistance develops, it could spread rapidly, nullifying the intervention and causing the loss of the driving construct [110–113]. In addition to this molecular resistance, insects may evolve other strategies to bypass the effects of engineered symbionts. For example, pests might develop mechanisms to neutralize or suppress the toxic effects of symbiotic bacteria or alter their microbiome to exclude or outcompete engineered strains. Addressing these issues is crucial for the successful implementation of insect–microbe symbiosis-based pest-control strategies.

## Future directions

Precision in genetic engineering will be crucial for developing symbiotic microorganisms that specifically target pest species while minimizing unintended effects. Advances in understanding the molecular interactions between insects and their symbionts will be vital. Leveraging technologies such as CRISPR/Cas9 for precise genetic modifications can increase the specificity of pest control by these bacteria, improving their effectiveness in targeting pest species without impacting beneficial organisms or disrupting ecosystems. By modifying symbiotic bacteria to express anti-pathogen compounds or dsRNA, the RNA interference pathway can be activated in insects, protecting them from viral attacks and controlling the spread of viruses by pests. This strategy has been demonstrated with *Snodgrassella alvi*, a symbiotic gut bacterium of bees that can be genetically manipulated to induce eukaryotic RNAi immune responses, thereby altering bee physiology, behavior, and growth [70]. Future research could also explore multi-pronged approaches, such as designing microbial consortia that combine different strains of symbionts, each targeting specific pest-related pathways or diseases [114, 115]. Additionally, advancements in synthetic biology could lead to the design of "smart" microorganisms that can adapt to evolving pest populations or environmental changes, maintaining efficacy over the long term [116–118]. One promising avenue might be to use CRISPR/Cas9 systems for on-demand gene activation or repression, allowing bacteria to respond dynamically to the presence of pests or pathogens.

Non-target effects are a critical issue in genetic modification and microbial applications, particularly concerning potential cascading reactions in ecosystems. Therefore, long-term ecological monitoring is key to ensuring that non-target effects do not have negative impacts on the environment or biodiversity. This monitoring should not only focus on observing non-target species but also track the spread and stability of genetically modified microbes in natural environments. Additionally, through precise pest control strategies, such as the fine-tuned regulation of gene-editing technologies, the impact on non-target species can be minimized, ensuring the specificity and safety of pest control efforts. This approach can effectively reduce unnecessary ecological risks while enhancing the efficiency and sustainability of biological control methods.

Furthermore, more in-depth studies on microbial‒host interactions could reveal new molecular targets that can be harnessed to modify bacterial behavior in ways that directly impact pest physiology. Finally, long-term field trials will be critical to assess the real-world effectiveness of these genetically engineered symbionts, monitor for any ecological disruptions, and evaluate the potential for resistance development in pest populations. By focusing on precision genetic modifications and exploring these advanced technologies, future research could greatly increase the effectiveness of symbiosis-based pest control methods, providing a sustainable and eco-friendly alternative to traditional chemical pesticides.

Ensuring the effectiveness of symbiotic bacteria in various environments is crucial for the success of symbiosis-based pest control strategies. Previous studies have shown that biotic and abiotic factors affect symbiotic microorganisms [119–121]. For example, the symbiotic bacteria in the gut of the green vegetable bug *Nezara viridula* are sensitive to high temperatures, which may inhibit their activity [11]. These strains, which are highly affected by environmental factors, may face numerous limitations in pest control applications, even if they possess other advantageous traits. Further research should focus on developing bacterial strains that can endure environmental stress, such as temperature fluctuations, humidity, and exposure to insecticides. These strategies aim not only to increase the stability and persistence of symbiotic microorganisms but also to improve their effectiveness in controlling pests under varying environmental conditions.

An integrated pest management approach is essential for maximizing the potential of insect–microbe symbiosis-based strategies. This involves combining this approach with other pest-control strategies to create a comprehensive and sustainable management system. For example, integrating symbiotic microorganisms with biological control agents, such as natural predators or parasitoids, can provide a multi-layered approach to pest management. Additionally, incorporating cultural practices such as crop rotation, habitat manipulation, and proper sanitation can further increase the effectiveness of these symbiotic microbe agents. The selective use of chemical pesticides in combination with insect-microbe symbiosis-based strategies may minimize environmental impact and prevent resistance development. By leveraging such diverse sets of methods, we can achieve more robust and resilient pest management solutions that balance effectiveness with ecological sustainability.

## Data Availability

Not applicable.
