# Correction: Insect‒microbe symbiosis-based strategies offer a new avenue for the management of insect pests and their transmitted pathogens

**DOI:** 10.1007/s44297-025-00041-8

**Published:** 2025-02-18

**Authors:** Chao Lv, Yan‑Zhen Huang, Jun‑Bo Luan

**Affiliations:** Liaoning Key Laboratory of Economic and Applied Entomology, College of Plant Protection, Shenyang Agricultural University, Shenyang, 110866 China

**Correction** **:** **Crop Health 2, 18 (2024)**

**https://doi.org/10.1007/s44297-024-00038-9**

Following publication of the original article [1], it is reported that the HTML version of Fig. 1 was incomplete. The error was caused by production during the figure conversion process, which led to the incomplete display of HTML version of Fig. 1.

The HTML version of Fig. 1 is corrected from: 

Fig. 1 Distribution of pest control strategies based on insect‒microbe symbiosis


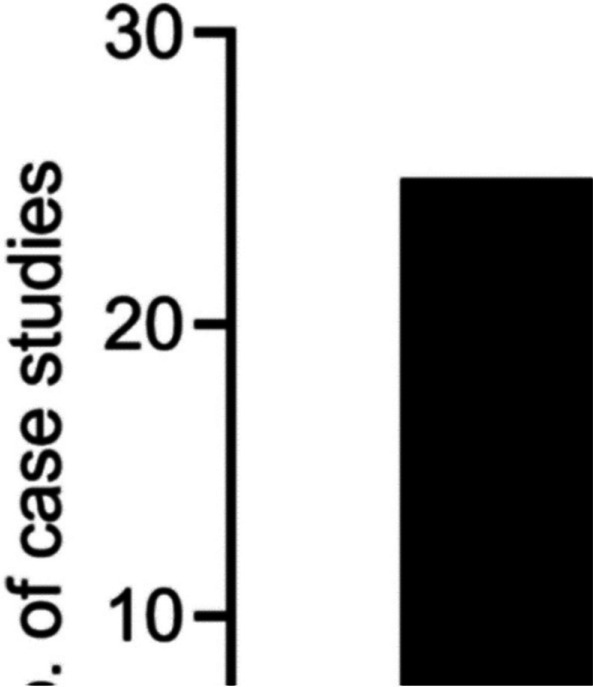


To:

Fig. 1 Distribution of pest control strategies based on insect‒microbe symbiosis


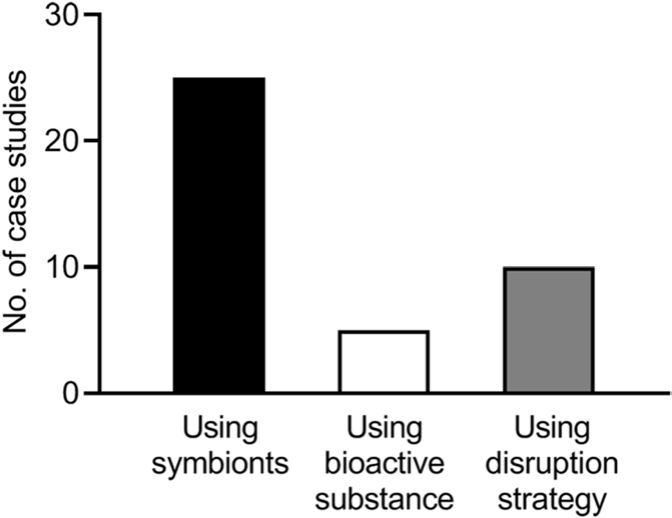


The original article [1] has been updated.
